# Analysis of orbital malignancies presenting in a tertiary care hospital in Pakistan

**DOI:** 10.12669/pjms.331.12073

**Published:** 2017

**Authors:** Asad Aslam Khan, Suhail Sarwar, Mohammad Ali A Sadiq, Imran Ahmad, Nayab Tariq, Sibghat-ul-Noor

**Affiliations:** 1 Prof. Dr. Asad Aslam Khan, MS (Ophth), PhD (Ophth). Institute of Ophthalmology/College of Ophthalmology and Allied Vision Sciences, King Edward Medical University, Mayo Hospital, Lahore, Pakistan; 2 Dr. Suhail Sarwar, MS Ophthalmology. Institute of Ophthalmology/College of Ophthalmology and Allied Vision Sciences, King Edward Medical University, Mayo Hospital, Lahore, Pakistan; 3 Dr. Mohammad Ali A Sadiq, FCPS Ophthalmology, FPOS (UK), FAAPOS (USA). Institute of Ophthalmology/College of Ophthalmology and Allied Vision Sciences, King Edward Medical University, Mayo Hospital, Lahore, Pakistan; 4 Dr. Imran Ahmad, DOMS, MPH. Institute of Ophthalmology/College of Ophthalmology and Allied Vision Sciences, King Edward Medical University, Mayo Hospital, Lahore, Pakistan; 5 Dr. Nayab Tariq, MBBS. Institute of Ophthalmology/College of Ophthalmology and Allied Vision Sciences, King Edward Medical University, Mayo Hospital, Lahore, Pakistan; 6 Dr. Sibghat-ul-Noor, MBBS. Institute of Ophthalmology/College of Ophthalmology and Allied Vision Sciences, King Edward Medical University, Mayo Hospital, Lahore, Pakistan

**Keywords:** Lymphoma, Malignancy, Orbit, Retinoblastoma, Rhabdomyosarcoma, Squamous cell carcinoma

## Abstract

**Objective:**

To determine the frequencies of various orbital malignancies amongst orbital lesions in patients presenting in a tertiary care hospital in Pakistan.

**Methods:**

A retrospective analysis of 666 orbital cases with an established histopathological diagnosis of malignant tumors treated in Mayo Hospital Lahore from 1996 to 2015 (20 years).

**Results:**

About 66% of the malignant tumors were primary, 25% secondary, 8% haematopoietic and 1% metastatic. Almost 50% of the cases were children. Retinoblastoma is the commonest tumor (43% overall and 87% among children). Squamous cell carcinoma is the second commonest (15.6% overall and 31% among adults). These are then followed by Adenoid cystic carcinoma of Lacrimal Gland (9%), Lymphoma/Leukaemia (8%) and Rhabdomyosarcoma (6.3%).

**Conclusion:**

Frequencies of various orbital malignancies show geographical variation in both paediatric and adult population.

## INTRODUCTION

Orbital malignancies are an important group of disorders presenting in all age groups. Malignant tumors of the orbit may originate either from the primary orbital tissues including the eyeball, or may invade from surrounding structures like eyelids, paranasal sinuses, nasopharynx or cranial cavity. They may also be a presentation of systemic lymphoproliferative disorders or metastasis from malignant tumors elsewhere in the body.^1^

Prevalence of the malignant tumors can show racial and geographical variation. For example, a study carried out in Denmark shows the most common malignant tumor of the orbit to be lymphoma, whereas a study carried out in China indicates Malignant Lacrimal gland tumors to be the commonest.^2^,^3^

Our study aims to find out the relative frequencies and age distribution of various malignant orbital tumors and compare their geographical variation. This can help to determine the trend of these tumors in this part of the world. This basic information can be useful in relating these tumors to certain racial and environmental factors.

## METHODS

A record of 1454 patients presenting with orbital lesions to Ophthalmology department of King Edward Medical University/Mayo Hospital Lahore from 1996 to 2015 was analyzed and 666 patients with established histopathological diagnosis of malignant orbital tumors were included in the study. After the diagnosis of malignancy was established, the patients were referred to the Oncology department for further management. The tumors were broadly classified into Primary, Secondary, Haematopoeitc and Metastatic.

## RESULTS

A total of 1454 cases of orbital diseases were analyzed over a period of 20 years. Out of these, 788 (54%) were benign and 666 (46%) cases were malignant. The frequencies of these 666 malignant tumors are shown in Table-I. 330 cases were children (up to 10 years) and 336 were adults (above 10 years). The tumors were classified as Primary (arising from the contents of the orbit including eyeball), Secondary (arising from the structure in the neighborhood of orbit like eyelids, para nasal sinuses, Cranial cavity and Nasopharynx), Hematopoietic reticuloendothelial system lesion and Metastatic tumours. 66% of the malignant tumors were primary in origin, 25% were secondary, 8% were haematopoeitic and 1% were metastatic.

**Table-I:** Percentage and age-wise breakup of all malignant orbital tumors

Tumor	Frequency	Up to 10 years of age	Above 10 years of age	%age of total malignancies
Total	666	330	336	
Retinoblastoma	291	287	4	43.69
Squamous cell carcinoma of eyelid	104	-	104	15.62
Lymphoma/Leukaemia	53	9	44	7.96
Rhabdomyosarcoma	42	29	13	6.31
Adenoid cystic carcinoma of Lac. gland	35	-	35	5.25
Adenocarcinoma of lacrimal gland	25	2	23	3.75
Maxillary antrum carcinoma	18	-	18	2.70
Basal cell carcinoma	18	-	18	2.70
Sebaceous cell carcinoma eyelid	16	-	16	2.40
Choroidal melanoma	16	-	16	2.40
Lacrimal gland Lymphoma	15	-	15	2.25
Malignant melanoma eyelid	7	-	7	1.05
Soft tissue sarcoma	6	1	5	0.90
Metastatic	6	1	5	0.90
Osteosarcoma	4	-	4	0.60
Haemangiopericytoma	2	-	2	0.30
Acinic cell CA	2	1	1	0.30
Malignant Sebaceous cyst	2	-	2	0.30
Mucoepidermoid CA	1	-	1	0.15
Adenocarcinoma Meibomian gland	1	-	1	0.15
Plasmacytoma	1	-	1	0.15
Malignant Angioma	1	-	1	0.15

The primary malignant tumors were classified based on their origin as ocular, lacrimal gland, vascular, muscular, osseous and connective tissue. (Table-II, Table-III,)

**Table-II:** Frequencies of primary malignant orbital tumors.

Tumors	Total	Male	Female	%age out of primary malignant	%age out of total malignant
***Ocular***					
Retinoblastoma	299	183	108	66.14	43.69
Choroidal Melanoma	16	11	5	3.64	2.40
***Lacrimal Gland***					
Adenoid cystic carcinoma	35	9	26	7.95	5.25
Adenocarcinoma	25	10	15	5.68	3.75
Lacrimal gland lymphoma	15	6	9	3.41	2.25
Acinic cell carcinoma	2	2	-	0.45	0.30
Mucoepidermoid carcinoma	1	1	-	0.23	0.15
***Muscular***					
Rhabdomyosarcoma	42	24	18	9.55	6.31
***Soft tissue***					
Sarcomas	6	3	3	1.36	0.90
***Osseous***					
Osteosarcoma	4	3	1	0.91	0.60
***Vascular***					
Malignant haemangiopericytoma	2	2	-	0.45	0.30
Malignant angioma	1	1	-	0.23	0.15

**Table-III:** Frequencies of primary malignant orbital tumors in different age groups.

Tumors	Total	<10 years	10- <20 years	20 - <40yrs	40 - <60yrs	60 and above	Mean Age (yrs)
***Ocular***							
Retinoblastoma	291	287	4	-	-	-	3.5
Choroidal melanoma	16	-	1	5	8	2	45
***Lacrimal Gland***							
Adenoid cystic carcinoma	35	-	3	26	5	1	31
Adenocarcinoma	25	2	1	15	3	4	40
Lacrimal gland lymphoma	15	-	-	5	6	4	49
Acinic cell carcinoma	2	1	1	-	-	-	7
Mucoepidermoid carcinoma	1	-	-	1	-	-	27
***Muscular***							
Rhabdomyosarcoma	42	29	11	2	-	-	11
***Soft tissue***							
Sarcoma	6	1	1	1	1	2	43
***Osseous***							
Osteosarcoma	4	-	2	2	-	-	27
***Vascular***							
Malignant haemangiopericytoma	2	-	-	1	1	-	37.5
Malignant angioma	1	-	-	-	1	-	40

Out of the 307 Malignant Ocular tumors, 291 (94.78%) cases were retinoblastomas (183M, 108F), ranging in ages between 18 days to 10 years (Mean Age=3.5y) and 16 (5.21%) were choroidal melanomas (11F and 5M) presenting in ages 16 to 75 years (Mean Age=45y).

Out of 78 malignant Lacrimal gland tumours, 35 (9M, 26F) were Adenoid cystic carcinoma presenting in ages 14 to 75 years (Mean Age=31y), 25 (10M, 15F) were Adenocarcinoma with age range 4 to 72 years (Mean Age=40y), 15 were Lacrimal gland lymphoma (6M, 9F) with age range of 20 to 65 years (Mean Age=49y), 2 cases (2M) were of Acinic cell carcinoma (2.56%) aged 1.5 and 12 years (Mean Age=7y), and one case (F) of Mucoepidermoid carcinoma (1.28%) aged 27 years.

Out of the remaining primary tumors, 42 (24M, 18F) were muscular in origin, all of which were Rhabdomyosarcomas presenting in ages 1.5 to 70 years (Mean Age=11y), 6 (3M, 3F) were soft tissue sarcomas with ages between 15 and 75 years (Mean Age=43y), 4 (3M, 1F) osteosarcomas, ages 20 to 45 years (Mean Age=27y) and 3 vascular in origin; 2 (M) Malignant Haemangiopericytoma, ages 35 and 40 years and 1 (M) Malignant Angioma with age 40 years.

Among the 257 secondary orbital lesion, 91 (35.4%) were benign and 166 (64.5%) were malignant. The most common source of the secondary malignant orbital tumors was eyelid, in 148 cases (89.1%) as opposed to paranasal sinuses in 18 cases (10.9%). Commonest tumor in this category was Squamous cell carcinoma of eyelid 104 (60M, 44F) cases presenting in ages 9 to 110 years (Mean Age=51y) (Table-IV). Other eyelid tumors were Basal cell carcinoma 18 (7M, 11F) with age range 33 to 70 years (Mean Age=64y), Sebaceous cell carcinoma 16 (12M, 4F), age range 40 to 75 years (Mean Age=64y), Malignant melanoma of eyelid 7 (5M, 2F), age range 30 to 72 years (Mean Age=59y), Malignant sebaceous cyst 2 (F) both 60 years, and Adenocarcinoma of Meibomian gland 1 (M), age 55 years. 18 (12M, 6F) cases were of Maxillary antrum carcinoma, age range 28 to 70 years (Mean Age=61y) (Table-V).

**Table-IV:** Frequencies of secondary malignant orbital tumors.

Tumors	Total	Male	Female	%age of secondary malignant	%age of total
***Eyelid***					
Squamous cell carcinoma	104	64	44	62.65	15.62
Basal cell carcinoma	18	7	11	10.84	2.70
Sebaceous cell carcinoma	16	12	4	9.64	2.40
Malignant melanoma	7	5	2	4.22	1.05
Malignant sebaceous cyst	2	-	2	1.20	0.30
Adenocarcinoma meibomian gland	1	1	-	0.60	0.15
***Paranasal sinuses***					
Maxillary Antrum carcinoma	18	12	6	10.84	2.70

**Table-V:** Frequencies of secondary malignant orbital tumors in different age groups.

Tumors	Total	<10 yrs	10 - <20yrs	20 - <40 yrs	40 - <60 yrs	60 yrs and above	Mean Age (yrs)
***Eyelid***							
Squamous cell carcinoma	104	-	7	12	31	54	51
Basal cell carcinoma	18	-	-	1	9	8	64
Sebaceous cell carcinoma	16	-	-	-	5	11	64
Malignant melanoma	7	-	-	1	1	5	59
Malignant sebaceous cyst	2	-	-	-	1	1	60
Adenocarcinoma meibomian gland	1	-	-	-	1	-	55
***Paranasal sinuses***							
Maxillary Antrum carcinoma	18			5	7	6	61

Orbital involvement as a part of malignant reticuloendothelial/lymphoproliferative disorders was found in 53 (26M, 27F) cases of lymphomas/leukaemias presenting in ages 3.5 years to 65 years (Mean Age=37y) and 1 (M) case was diagnosed as Plasmacytoma, age 42 years. Table-VI shows age distribution for these tumors.

**Table-VI:** Frequencies of RES/lymphoproliferative disorders in different age groups.

Tumors	Total	<10 yrs	10 - <20 yrs	20 - <40 yrs	40 - <60 yrs	60 yrs and above	Mean Age (yrs)
Lymphoma/Leukaemia	53	9	3	11	23	7	37
Plasmacytoma	1	-	-	-	1	-	42

**Metastasis** in orbit from other sources was found in 6 cases, 4 (2M, 2F) of which came out to be Ewing’s sarcoma, ages 10 to 28 years (Mean Age=17y) and in 2 cases primaries could not be established.

## DISCUSSION

Various studies conducted on orbital malignancies have shown differences in their relative frequencies in different areas of the world. The present study serves to add to the already existing worldwide data and to compare the results.

The statistics of this study show 54% of the tumors to be benign and 46% to be malignant. This observation of benign lesions being greater than the malignant tumors is consistent with other studies like the ones conducted in Dublin and India.^4^,^5^ The study from Dublin however reports secondary tumors to be the commonest source whereas the present study reports primary orbital tissue to be the most common source of orbital tumors.^4^ Studies from Nepal and Brazil also reported eyelid as the commonest source of malignant orbital tumors as compared to Retinoblastoma in the present study, they however reported Sebaceous cell carcinoma and Basal cell carcinoma to be the commonest respectively, as compared to squamous cell carcinoma in this study.^6^,^7^

In the present study, the overall commonest malignant tumor was Retinoblastoma (43.69%), although it is exclusively a tumor of paediatric age group. Retinoblastoma was followed by Squamous cell carcinoma of the eyelid (15.62%) which was found to be the most common tumor of the adults, followed by adenoid cystic carcinoma of the lacrimal gland (9%), and lymphomas/leukaemias (8%). However, a study conducted in China reports lacrimal gland to be the commonest source of malignant tumors and studies conducted in Netherlands and Florida reported Lymphomas as the most common malignant tumors.^2^,^8^,^9^ The study from India reported Squamous cell carcinoma to be the commonest.^5^ A study carried out in Larkana, Pakistan reports equal prevalence of Lymphomas, Squamous cell carcinoma and Retinoblastoma.^10^

Among children, the second most prevalent tumor after Retinoblastoma (mean age=3.5y, 87% of children) was Rhabdomyosarcoma (mean age=11y, 8.7% of children), however it was also found in patients till 3rd decade of life. A study by Modi (India) also reports retinoblastoma as the commonest malignant tumor in children but the results hugely differ from the studies conducted by Gunalp and Gunduz (Turkey) and Koopman (Netherlands) who have reported rhabdomyosarcoma to be the commonest tumor among children.^8^,^11^,^12^

Exclusively found among the adults, Squamous cell carcinoma of eyelid was the most frequently occurring tumor of the adult age group (mean age=51y, 31% of adults), followed by Adenoid cystic carcinomas (mean age=31.5y, 17.3% of adults) and Lymphomas (mean age=37y, 13.1% of adults). Demicri however, has reported Lymphomas as the most frequently occurring malignant tumors in adults.^13^

## CONCLUSION

The above discussed differences show that there exists a geographical variation in the frequency of various tumors. This difference may be due to over presentation of certain tumors related to specific age group in our tertiary care hospital. However, as our study reports, Retinoblastoma should be kept on top of the list while making differentials in paediatric age group. Reporting these differences in frequencies of various tumors can help a clinician in making a diagnosis. Moreover, this study gives way to establishing the association of geographical factors with development of certain malignancies.

## References

[1] Khan AA, Sarwar S, Sadiq AAM, Rehman MU, Ullah A, Ahmad I (2015). Analysis of 1246 cases of orbital lesions: A study of 17 years. Natural Science.

[2] Ni C (1991). Histopathologic classification of 1422 orbital tumors. Zhonghua Yan Ke Za Zhi.

[3] Johansen S, Heegaard S, Bogeskov L, Prause JU (2000). Orbital space-occupying lesions in Denmark 1974-1997. Acta Ophthalmologica.

[4] Shields JA, Shields CL, Scartozzi R (2004). Survey of 1264 patients with orbital tumors and simulating lesions: The 2002 Montgomery Lecture, part 1. Ophthalmology.

[5] Shaikh IY, Shah FR, Gandhi MB, Shah CK, Shah NR (2012). Ophthalmic Neoplastic Lesions –A Retrospective study of 4 years. Gujarat Med J.

[6] Bastola P, Koirala S, Pokhrel G, Ghimire P, Adhikari RK (2013). A Clinico-histopathological study of Orbital and Ocular lesions;A Multicentre study. J Chitwan Med Coll.

[7] Sirianni D, Leles CR, Mendonca EF (2013). A 12-Year Retrospective Survey of Management of Patients with Malignant Neoplasms in the Orbital Cavity in a Brazilian Cancer Hospital. Open Dent J.

[8] Koopman JH, Loo MH, Dijk MR, Bijlsma WR (2011). Incidence of primary malignant tumors in Netherlands. Eye.

[9] Margo CE, Mulla ZD (1998). Malignant tumors of orbit. Analysis of the Florida cancer registry. Ophthalmology.

[10] Halepota FM, Talpur KI, Luhano MK, Shaikh LSM (2014). Orbital Tumors –Retrospective study of 24 years. Pak J Ophthalmol.

[11] Gunalp I, Gunduz K (1994). Biopsy-proven orbital lesions in Turkey: A survey of 1092 cases over 30 years. Orbit.

[12] Modi PJ, Shah NA, Bhalodia JN, Gonsai RN (2013). Orbital tumors in children: A descriptive study at tertiary care centre. NJMR.

[13] Demicri H, Shield CL, Shields JA, Honavar SG, Mercado GJ, Tovilla JC (2002). Orbital tumors in older adult population. Ophthalmology.

